# Monitoring the Milk Composition, Milk Microbiota, and Blood Metabolites of Jersey Cows throughout a Lactation Period

**DOI:** 10.3390/vetsci10030226

**Published:** 2023-03-16

**Authors:** Peter Kiiru Gathinji, Zabiallah Yousofi, Karin Akada, Ajmal Wali, Naoki Nishino

**Affiliations:** 1 Department of Animal Science, Graduate School of Environmental and Life Science, Okayama University, Okayama 700-8530, Japan; 2 Animal Products Research Group, Institute of Livestock and Grassland Science, National Agriculture and Research Organization, Ibaraki 305-0901, Japan

**Keywords:** airborne dust, blood metabolites, Jersey cows, microbiota, milk

## Abstract

**Simple Summary:**

The shift to the sustainable production of quality milk has led to the increased breeding of Jersey cows. This study examined the milk composition, milk microbiota, and blood metabolites throughout the lactation period of Jersey cows, producing a peak milk yield of more than 25 kg/d. The prevalent families of the milk microbiota changed intermittently during the lactation period. Contamination of the environmental microbiota in milk was coupled with elevated plasma non-esterified fatty acids, haptoglobin, and aspartate transaminase levels. Thus, impaired metabolic function during the early lactation period may increase the invasion of opportunistic bacteria.

**Abstract:**

This study aimed to determine how milk composition, milk microbiota, and blood metabolites may change during the lactation period in Jersey cows. Milk and jugular blood samples were collected from eight healthy cows every other month from the beginning to the end of their lactation period. Samples of airborne dust were also collected to determine whether the cowshed microbiota could affect milk microbiota. Milk yield peaked in the first two months and gradually decreased as the lactation period progressed. Milk fat, protein, and solids-not-fat contents were low in the first month, and then increased during the middle and late lactation periods. In the first month, plasma non-esterified fatty acids (NEFA), haptoglobin (Hp), and aspartate transaminase (AST) levels were elevated, and high abundances of Burkholderiaceae and Oxalobacteraceae were observed in milk and airborne dust microbiota. The finding that contamination of the environmental microbiota in milk was coupled with elevated plasma NEFA, Hp, and AST levels indicated that impaired metabolic function during the early lactation period may increase the invasion of opportunistic bacteria. This study can affirm the importance of feeding and cowshed management and should provide a helpful addition to improving Jersey cow farming.

## 1. Introduction

Owing to the sustained demand for milk, Holstein cows have been the most reared breed globally. This unmatched milk production usually causes Holstein cows to experience a rather intensive negative energy balance after calving. Holsteins reportedly have a lower conception rate than other breeds of cows, and the days open and the number of services are greater for Holsteins than Jersey cows [1,2,3,4]. These facts, coupled with lower milk components produced by Holsteins compared to other breeds, have gradually galvanized dairy stakeholders to seek alternative breeds, such as Jerseys.

Milk produced by Jersey cows has been gaining traction from farmers and manufacturers due to its higher fat and protein contents [5,6,7]. Jersey milk fetches higher prices for farmers and has superior characteristics for processing dairy products. Differences in productivity and milk quality between Jersey and Holstein cows may lead to different microbial profiles in milk. Tardon et al. [8] reported significant associations between blood energetic metabolites (non-esterified fatty acids (NEFA), triglycerides, and cholesterol) and colostrum microbiota in Holstein cows. Metabolites indicating protein status, that is, total protein and albumin, were not related to colostrum microbiota in the study. Janovick Guretzky et al. [9] observed lower levels of plasma NEFA in Jerseys than in Holsteins ten days postpartum, whereas Brown et al. [10] found no differences in NEFA levels ten weeks postpartum between the two breeds. Mastitis, an inflammation caused by increased pathogens and microbiota dysbiosis in milk, occurs more frequently in Holsteins than in Jerseys [3], although the opposite observation has also been reported [10]. It is still unclear how such metabolite fluctuations influence the milk microbiota of Jersey cows. Unlike the manifold research that has focused on lactation-long microbial changes in milk produced by Holsteins, few studies have investigated changes in the milk microbiota of Jersey cows in relation to metabolic profiles across the lactation period.

This study aimed to investigate how the microbiota of milk produced by Jersey cows may change during the entire lactation period, and whether the milk microbiota changes are related to blood metabolite levels, indicating the balance of nutrients. The airborne dust microbiota in the cowshed was also investigated to examine its association with milk microbiota. 

## 2. Materials and Methods

### 2.1. Sample Collection 

Milk samples were collected from eight lactating Jersey cows reared at Chugoku Shikoku College of Dairy Farming, located in the Hiruzen region of Okayama Prefecture, Japan. Cows that calved during the same month (August) as their first or second parturition were selected. Milk samples were collected in the first month (M1) and every other month until the end of the lactation period (M2, M4, M6, M8, and M10). The Jersey farm managed lactating cows in free-stall barns and offered two total mixed rations that differed in rumen undegradable protein and starch content (Table 1). Based on the milk yield and composition of the individual cows obtained from the monthly milk test, the herd was assigned to high- or low-yielding cows, in addition to the stage of lactation. The cows were milked twice daily at a parlor and morning milking samples were collected. The first few streams of foremilk were discarded after cleaning the surface of the four teats. Subsequent streams from the four teats were collected in a 50 mL collection tube, and the composite milk was frozen in liquid nitrogen. Frozen milk was stored at −30 °C until further analysis. 

After milking from 05:30 to 07:00, the cows gained access to the newly provided feed. Blood samples were collected from 08:30 to 09:30 from the jugular vein, stored in heparinized tubes, and transported on ice. Airborne dust samples were collected from the cowshed by placing five open Petri dishes (90 × 14 mm) at different locations approximately one meter above the ground for approximately five minutes [11]. After the collection time had elapsed, each Petri dish was washed with 1 mL of sterile saline to prepare a composite sample. The composite sample was frozen in liquid nitrogen and stored at −30 °C until DNA extraction. The number of milk and airborne dust samples were, thus, eight and one on each sampling day, respectively. All procedures and protocols for animal experiments were approved by the Animal Care and Use Committee, Okayama University (OKU-2020856), Japan.

### 2.2. Milk Composition and Blood Metabolites Analyses

The levels of fat, protein, solids-not-fat (SNF), urea nitrogen (MUN), and somatic cell count (SCC) in the milk were determined using a CombiFoss FT+ analyzer (Foss Allé, Hillerød, Denmark). The plasma concentrations of total cholesterol (T-Cho), NEFA, albumin (Alb), urea nitrogen (BUN), calcium (Ca), phosphorus (P), aspartate transaminase (AST), and alanine transaminase (ALT) were determined using commercially available kits (FUJIFILM Wako Pure Chemicals Co., Tokyo, Japan). Plasma haptoglobin (Hp) concentration was determined using a commercial ELISA kit (Life Diagnostics, Inc., West Chester, PA, USA).

### 2.3. Bacterial DNA Extraction

Bacterial DNA was extracted from the milk and airborne dust samples and purified as described by Nguyen et al. [12]. One milliliter (1 mL) of milk was centrifuged at 16,000× *g* for 15 min at 4 °C. The fat layer was scraped off and the supernatant was removed. One milliliter (1 mL) of the composite airborne dust sample was centrifuged under the same conditions. The DNA pellets were washed and resuspended in 1 × PBS (phosphate-buffered saline) and the resuspended DNA pellets were transferred into 2 mL screw-cap tubes containing 0.4 g zirconia beads (0.3 g of 0.1 mm and 0.1 g of 0.5 mm) and 1 mL of lysis buffer (0.5 M NaCl, 0.05 M Tris-HCl, 0.05 M EDTA, 4% sodium dodecyl sulfate). The mixture was homogenized using a mini-bead beater. DNA was purified using a DNeasy Stool Mini Kit (Qiagen, Germantown, MD, USA).

### 2.4. 16S rRNA Gene Amplicon Sequencing

The primers targeting the V4 region of 16S ribosomal RNA (rRNA) genes (forward: 5′-ACACTCTTTCCCTACACGACGCTCTTCCGATCTGTGCCAGCMGCCGCGGTAA-3′; reverse: 5′-GTGACTGGAGTTCAGACGTGTGCTCTTCCGATCTGGACTACHVGGGTWTCTAAT-3′; tail sequences are underlined) were used for the first-round PCR [13], with the following protocol: initial denaturation at 94 °C for 2 min, followed by 35 cycles of denaturation at 94 °C for 30 s, annealing at 50 °C for 30 s, elongation at 72 °C for 30 s, and an elongation step at 72 °C for 5 min. The PCR products were electrophoresed on a 1% agarose gel and purified using a Fast Gene Gel/PCR Extraction Kit (NIPPON Genetics Co., LTD., Tokyo, Japan). The second-round PCR, with adapter-attached primers, followed the protocol of initial denaturation at 94 °C for 2 minutes, 10 cycles of denaturation at 94 °C for 30 s, annealing at 59 °C for 30 s, elongation at 72 °C for 30 s, and an elongation step at 72 °C for 5 minutes. The PCR products were purified in the same way as the case of the first-round PCR products. The purified amplicons were subjected to MiSeq sequencing at FASMAC Co., Ltd (Kanagawa, Japan).

### 2.5. Bioinformatics and Statistical Analyses

Quantitative Insights into Microbial Ecology 2 was used to analyze the paired-end sequences. Raw sequences, which were approximately 250 bp, were truncated at the site with a quality score below 25, and the reads with a 60 bp overlap were joined. The quality-assessed, chimera-free, and paired-end sequences were grouped into operational taxonomic units (OTUs) with a similarity threshold above 97%. 

Data on milk composition, milk microbiota, and blood metabolites were analyzed by repeated measures of analysis of variance using JMP software (ver. 14.2, JMP Japan, Tokyo, Japan). Tukey’s multiple comparisons tests were used to determine the statistical differences between means. Principal coordinate analysis (PCoA) was performed using Primer7 (ver. 7, Primer-E, Plymouth Marine Laboratory, Plymouth, UK).

## 3. Results

### 3.1. Milk Yield, Milk Composition, and Blood Metabolites Concentration

The average milk yield was 22.6 kg/d during the lactation period. The yield peaked at about 27.0 kg/d at M1 and M2 and then gradually decreased as the lactation period progressed (Table 2). The average milk yield at M10 was 14.8 kg/d. Milk components appeared to become denser with a decline in milk yield. Although milk fat (4.49%), protein (3.39%), and SNF (9.01%) contents were low at M1, they all increased in the middle and late lactation periods. The highest fat (5.85%), protein (4.54%), and SNF (10.0%) contents were observed at M6 and M10. The SCC varied within threshold levels (<2.0 × 10^5^ cells/mL), and no significant differences were found during the lactation period. Changes in the MUN levels differed from the other milk components, and the level showed a nadir at M2 and a sharp increase at M6.

Plasma Alb, BUN, and Ca levels were sharply elevated at M6. The T-Cho level was lowest (160 mg/dL) at M1, peaked (229 mg/dL) at M2, and then progressively declined during the late lactation period. After parturition, the NEFA level was highest (0.39 mEq/L) at M1 and progressively decreased toward the late lactation period. Likewise, the Hp level gradually decreased, from 62.6 to 10.6 μg/L, as the lactation period progressed. The Ca and P levels fluctuated throughout the lactation period with averages of 8.88 and 4.80 mg/dL, respectively. AST and ALT levels also fluctuated across the lactation period, and high levels were seen at M1 and M4 for AST and at M4 and M6 for ALT.

### 3.2. Milk Microbiota 

The α-diversity indices, Chao1 and Shannon, were highest at M1 but varied throughout the lactation period (Table 3). The lowest α-diversity was observed at M1 and M10.

**Figure 1:**
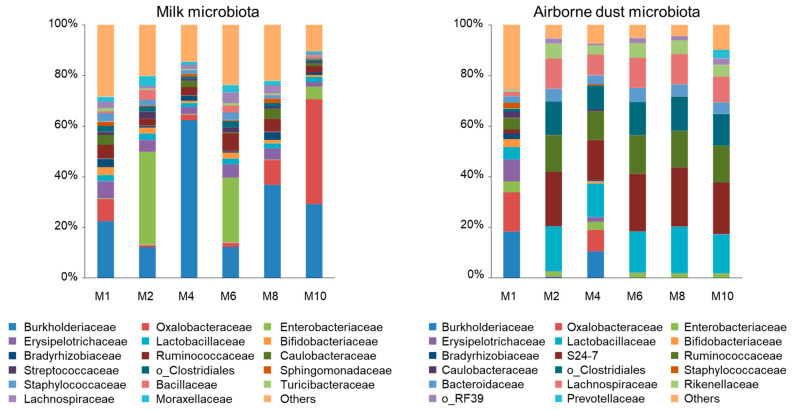
Family-level proportions of the microbiota of the milk of Jersey cows and the airborne dust microbiota of the cowshed. Bacterial taxa detected at >2% in at least one sample are indicated. Bars for milk microbiota indicate mean values of eight samples and those for airborne dust indicate the value of a single determination of the composite sample. M1, M2, M4, M6, M8, and M10 represent the first, second, fourth, sixth, eighth, and tenth months of lactation, respectively.

Regardless of the lactation period, Proteobacteria was the most (48.1–80.5%) and Firmicutes was the second most abundant (12.6–36.4%) phyla in the milk microbiota (Figure 1). At the family level, the most abundant taxa were Burkholderiaceae (22.4–62.3% at M1, M4, and M8), Enterobacteriaceae (25.8–36.8% at M2 and M6), and Oxalobacteraceae (41.4% at M10). In addition, these three families were the second most abundant taxa, except at M4, during lactation. Erysipelotrichaceae (1.90–6.70%) and Ruminococcaceae (2.55–7.09%) were the third and fourth most abundant taxa in most milk samples. Moraxellaceae (0.76–3.84%), Caulobacteraceae (0.16–4.05%), Bacillaceae (0.06–3.94%), and Lachnospiraceae (0.80–4.25%) were identified as the fourth and fifth most abundant taxa. Staphylococcaceae (0.52–3.27%) and Streptococcaceae (0.51–2.81%), to which typical mastitis pathogens belong, were found across the lactation period, but neither of them were included in the five most abundant taxa.

### 3.3. Airborne Dust Microbiota

Here, differences are described numerically because statistical analysis could not be performed for the airborne dust obtained as a single composite sample at each collection. In most of the airborne dust microbiota (M2, M4, M6, M8, and M10), S24-7 (15.8–23.4%), Lactobacillaceae (13.2–18.4%), and Ruminococcaceae (11.4–15.3%) were the first, second, and third most abundant families in the cowshed (Figure 1). Clostridiales (9.42–13.4%) and Lachnospiraceae (8.25–12.0%) were the fourth and fifth most abundant taxa, respectively. Burkholderiaceae (18.3%), Oxalobacteraceae (15.5%), and Erysipelotrichaceae (8.60%) were the three most abundant taxa at M1, followed by Lactobacillaceae (4.80%) and Ruminococcaceae (4.61%). Similarly, Burkholderiaceae (10.5%) was the fourth most abundant taxon at M4 in the cowshed. Enterobacteriaceae, which was found to be the most abundant taxa at M2 and M6 in milk, was detected at 1.63–4.44% and was not one of the five most abundant taxa in the airborne dust microbiota.

### 3.4. Relationship between Milk Composition, Blood Metabolite Concentrations, Milk Microbiota, and Airborne Dust Microbiota 

**Figure 2:**
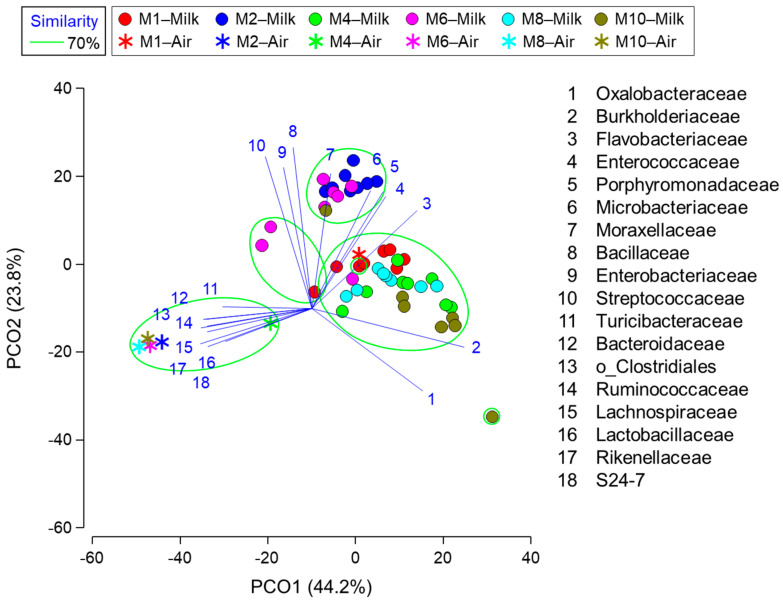
Principal coordinate plot characterizing the milk and airborne dust microbiota of the Jersey farm. Operational taxonomy units with Pearson’s correlations of >0.7 are overlaid on the plot as vectors. The samples enclosed by the green lines are considered in the same group at the similarity level of 70%. M1, M2, M4, M6, M8, and M10 represent the first, second, fourth, sixth, eighth, and tenth months of lactation, respectively.

The PCoA revealed that the milk microbiota collected at M2 and M6, which had Enterobacteriaceae as the most abundant taxon, were separately grouped from those collected at M1, M4, and M8, which had Burkholderiaceae as the most abundant taxon (Figure 2). Although the M10 samples had Oxalobacterceae as the most abundant taxon, the milk microbiota was grouped with the M1, M4, and M8 samples. Furthermore, the milk microbiota of one M1 and two M6 samples, which had a high abundance of Ruminococcaceae, formed a separate group.

Except for the M1 samples, the airborne dust microbiota were grouped separately from the milk microbiota. Bacterial taxa that featured the M2, M4, M6, M8, and M10 samples were Turicibacteraceae (1.29–1.74%), Bacteroidaceae (3.44–5.55%), and Rikenellaceae (3.55–5.86%), in addition to the five most abundant taxa (S24-7, Lactobacillaceae, Ruminococcaceae, Clostridiales, and Lachnospiraceae. The M1 sample had numerically lower abundances of Turicibacteraceae (1.09%), Bacteroidaceae (2.60%), and Rikenellaceae (0.58%).

**Figure 3:**
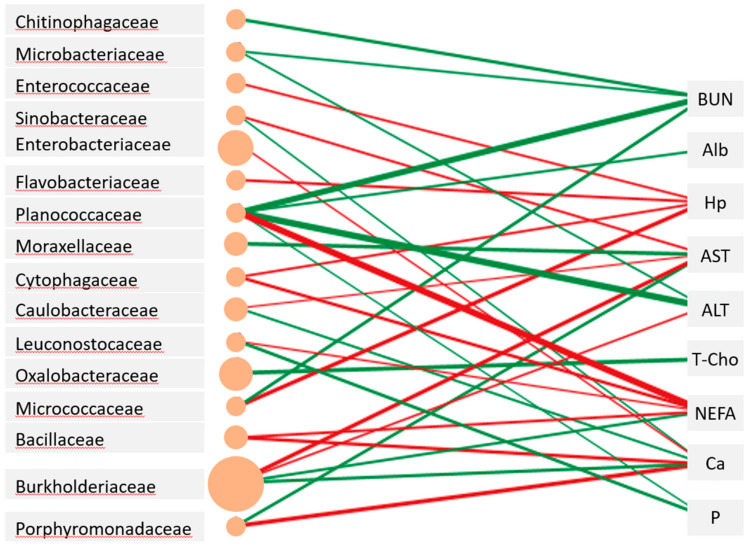
Networks showing the correlations (*p* < 0.01) between milk microbiota and blood metabolites of the Jersey cows. The edge width is proportional to the correlation coefficient between the abundance of bacterial family and the level of blood metabolites, and the size of the ellipse is proportional to the abundance of the bacterial family. Red and green colors depict positive and negative relationships, respectively.

The abundance of Burkholderiaceae in milk was positively correlated with plasma AST and ALT levels and that of Oxalobacteraceae in milk was negatively correlated with T-Cho levels (Figure 3). A distinctive positive relationship was observed between plasma NEFA levels and Planococcaceae abundance in milk. Meanwhile, the abundances of Ruminococcaceae, Lachnospiraceae, Erysipelotrichaceae, and Lactobacillaceae did not show significant relationships with any of the blood metabolites examined.

## 4. Discussion

In this study, the milk yield of Jersey cows peaked at approximately 27 kg/d in the first two months (M1 and M2) and then gradually decreased to 15 kg/d in the last month (M10) as lactation progressed. An increase in milk fat content during lactation was comparable to the results reported by Bainbridge et al. [14], who monitored Jersey cows and showed similar levels of milk yield in this study. The finding that milk components became denser with a decrease in milk yield toward the end of lactation also agreed with the findings of Bainbridge et al. [14]. The SCC levels did not show marked changes during lactation, except at M8, when the average SCC level was elevated beyond the threshold of 2.0 × 10^5^ cells/mL [15]. Two of the eight cows showed a high average SCC (6.0 × 10^5^ cells/mL); however, they did not show mastitis-related symptoms, and their milk microbiota was not markedly different from those of other cows.

MUN and BUN are indicators that help understand whether rumen degradable and undegradable proteins meet the demand of lactating cows [16,17]. Gustafsson and Palmquist [18] found that MUN equilibrates with BUN, and a similar observation was made in this study. Elevations in MUN and BUN levels at M6 could be due to a change in the TMR fed to cows. Milk production decreased by approximately 20% at M6 compared to M4; hence, the TMR may have changed from one for high milk yield to another for low milk yield with less rumen undegradable protein and starch contents. When sampled at M6, the cows were probably in the adaptation period for the altered nitrogen and energy balance in the rumen. Regardless, these changes did not accompany a marked increase or decrease in the concentrations of other metabolites.

An estimator of energy balance, NEFA, was expected to be higher at the beginning and to decrease as lactation progressed [19]. Similarly, although T-Cho was low at the beginning, the level increased when milk yield peaked, followed by a slight decline toward the end of lactation. These results imply that the cows had sufficient energy during the early and middle stages of lactation. During the periparturient period, lactating cows are more susceptible to reproductive and metabolic disorders. When the conversion of NEFA to triglycerides exceeds the metabolic capacity of hepatocytes, triglycerides accumulate, resulting in the impairment of liver function [20,21], which can be monitored by changes in blood AST and ALT levels [22]. Postpartum reproductive and metabolic disorders can also be monitored by measuring blood Hp levels [23,24]. Although plasma Hp levels were higher in the early lactation period than in the middle and late lactation periods in this study, the level was sufficiently low (<100 μg/L). The changes in metabolic profiles recorded in this study can serve as an example of healthy Jersey cow management.

Proteobacteria, rather than Firmicutes, predominated the milk microbiota throughout the lactation period in this study, despite many reports demonstrating that Firmicutes was the predominant phylum in the milk microbiota, regardless of healthy and mastitic udders [25,26]. Burkholderiaceae, Enterobacteriaceae, and Oxalobacteraceae were the most abundant families in three (M1, M4, and M8), two (M2 and M6), and one (M10) periods, respectively; hence, the families belonging to the phylum Proteobacteria varied during lactation. Metzger et al. [27] reported variations in the number of taxa in the milk microbiota, including Burkholderiaceae, during five months of lactation. However, the most notable taxon that varied in prevalence was Bacteroidetes in their study, which increased in the first month and then dramatically declined as lactation progressed. The abundance of Bacteroidetes was highest at M1, but not different from that at M2, M4, M6, and M8; hence, the reduction of Bacteroidetes in the milk microbiota during lactation was not observed in this study. Although Burkholderiaceae and Oxalobacteraceae belong to the order Burkholderiales and are detectable in diverse environmental habitats such as water, soil, and plants, they have not been considered the core microbiota of bovine milk [28]. Since our previous study detected Proteobacteria (mostly Pseudomanadaceae) as the prevalent phylum in the milk microbiota of Jersey cows at different farms [13], breed and regional differences may result in a high abundance of Proteobacteria.

Dahlberg et al. [29] pointed out that the evaluation of milk microbiota was hampered by background contamination, primarily Methylobacteriaceae. Similarly, Salter et al. [30] reported species belonging to Burkholderiaceae and Oxalobacteraceae as potential contaminants in the microbiota in low-biomass environments. Concurrent sequencing of the negative control samples was not performed in the microbiota analyses. Thus, the concern that a high abundance of Proteobacteria, especially Burkholderiaceae and Oxalobacteraceae, in the milk microbiota could be an outcome of contamination during DNA purification and sequence-based analyses cannot be ruled out. Regardless, the high abundance of Burkholderiaceae and Oxalobacteraceae in milk and airborne dust microbiota observed at M1 might indicate an occasional invasion of the cowshed microbiota into milk. Nguyen et al. [12] found that airborne dust microbiota were grouped with milk microbiota at some, but not all, sampling times. Microbial taxa regarded as the core bovine milk microbiota, such as Lactobacillaceae, Lachnospiraceae, and Moraxellaceae, were stable at relatively low abundances throughout the lactation period in this study.

The finding that the abundance of Burkholderiaceae and Oxalobacteraceae in milk was positively or negatively related to plasma AST, ALT, NEFA, and T-Cho levels suggested that impaired and improved metabolism would affect the risk of environmental bacterial contamination in milk. Tardon et al. [8] demonstrated that the abundance of Rhodocyclaceae and Bacillaceae in milk was positively related to plasma triglyceride levels, and that Rhodothermaceae was positively related to plasma T-Cho levels. Although the taxa of milk microbiota showing significant relations to blood energetic metabolites were different between their study and ours, the fact that no relationship was seen between the milk microbiota and metabolites indicating the protein status was the same. Thus, when cows suffer from a severe negative energy balance and undergo uterine recovery in the first month, the risk of invasion by opportunistic bacteria in milk may increase. However, the cows in this study were able to resist pathogenic bacterial infections. Further studies should be performed to clarify how cows with impaired metabolism are unable to prevent the growth of contaminating pathogenic bacteria.

## 5. Conclusions

The milk composition, milk microbiota, and blood metabolites of Jersey cows, producing a peak milk yield of more than 25 kg/d, were examined. Milk fat, protein, and SNF contents were low in the first month, and then increased during the middle and late lactation periods. During the early lactation period, the plasma NEFA, Hp, and AST levels were high; hence, similar to Holsteins, high-producing Jerseys may have impaired metabolic functions owing to a negative energy balance and recovery from uterine damage. The prevalent families of the milk microbiota changed intermittently during the lactation period, i.e., Burkholderiaceae, Enterobacteriaceae, and Oxalobacteraceae were predominant. One-month postpartum, high abundances of Burkholderiaceae and Oxalobacteraceae were found in milk and airborne dust microbiota. Thus, impaired metabolic function during the early lactation period may increase the invasion of opportunistic bacteria. Although this study can affirm the importance of feeding and cowshed management, the findings should be a helpful addition to improving Jersey cow farming.

## Figures and Tables

**Table 1:** Formulation and chemical composition of the total mixed ration offered to Jersey cows.

	TMR for High Milk Yield	TMR for Low Milk Yield
Diet ingredients		
Grass silage	301	359
Grass hay	26.2	28.6
Legume hay	13.1	14.3
Wet brewers’ grains	22.9	25.0
Soybean meal	23.1	25.2
Beet pulp	52.6	57.4
Compound feed	561	490
Chemical composition		
Crude protein	166	166
Rumen degradable protein	97.9	105
Ether extract	36.2	35.3
aNDFom	298	327
Starch	306	263
NE_L_ (Mcal/kgDM)	1.67	1.64

Proportion as g/kgDM unless otherwise noted. The compound feed consists mainly of corn and barley. aNDFom: ash-free neutral detergent fiber pretreated with α-amylase, NE_L_: net energy of lactation. Chemical composition was determined by near-infrared reflectance spectroscopy (Zen Raku Ren, Tokyo, Japan).

**Table 2:** Milk yield, milk composition, and blood metabolite levels of Jersey cows throughout the lactation period.

Lactation Period and Season at Sampling	M1 September	M2 October	M4 December	M6 February	M8 April	M10 June	SE
Milk yield (kg)	27.0 ^a^	27.3 ^a^	25.9 ^ab^	20.4 ^bc^	20.3 ^bc^	14.8 ^c^	0.67
Milk composition							
Fat (%)	4.49 ^c^	4.69 ^bc^	5.31 ^ab^	5.31 ^ab^	5.49 ^ab^	5.85 ^a^	0.08
Protein (%)	3.39 ^b^	3.47 ^b^	4.23 ^a^	4.54 ^a^	4.54 ^a^	4.46 ^a^	0.04
Solids-not-fat (%)	9.01 ^b^	9.20 ^b^	9.83 ^a^	10.0 ^a^	9.97 ^a^	9.83 ^a^	0.04
SCC (× 10^3^ cells/mL)	103	70.1	87.6	122	202	169	5.94
MUN (mg/dL)	8.96 ^c^	3.86 ^d^	8.71 ^c^	12.3 ^a^	10.3 ^bc^	11.6 ^ab^	0.20
Blood metabolites							
Albumin (g/dL)	3.85 ^c^	4.03 ^bc^	4.23 ^bc^	7.04 ^a^	4.33 ^b^	4.35 ^b^	0.04
BUN (mg/dL)	6.13 ^c^	5.57 ^c^	9.47 ^b^	14.2 ^a^	9.02 ^b^	10.6 ^b^	0.27
Total cholesterol (mg/dL)	160 ^b^	229 ^a^	214 ^ab^	209 ^ab^	196 ^ab^	180 ^ab^	5.65
NEFA (mEq/L)	0.39 ^a^	0.29 ^b^	0.11 ^c^	0.11 ^c^	0.09 ^c^	0.14 ^c^	13.59
Calcium (mg/dL)	8.28 ^bc^	9.25 ^ab^	8.15 ^c^	10.0 ^a^	8.94 ^abc^	8.61 ^bc^	0.10
Phosphorus (mg/dl)	3.43 ^b^	4.37 ^ab^	5.27 ^a^	4.52 ^ab^	5.45 ^a^	5.77 ^a^	0.15
AST (U/L)	112 ^a^	76.0 ^c^	116 ^a^	76.3 ^bc^	87.6 ^bc^	98.1 ^ab^	2.02
ALT (U/L)	16.5 ^c^	16.3 ^c^	33.6 ^a^	28.0 ^ab^	22.7 ^bc^	19.6 ^c^	0.63
Haptoglobin (μg/L)	62.6 ^a^	51.9 ^ab^	47.5 ^ab^	13.3 ^b^	15.3 ^b^	10.6 ^b^	3.68

Mean values for eight cows. Values in the same row with different superscript letters indicate statistically significant differences (*p* < 0.05). M1, M2, M4, M6, M8, and M10 represent the first, second, fourth, sixth, eighth, and tenth months of lactation, respectively. SCC: somatic cell count, MUN: milk urea nitrogen, BUN: blood urea nitrogen, NEFA: non-esterified fatty acid, AST: aspartate transaminase, ALT: alanine transaminase, SE: standard error.

**Table 3:** Microbiota diversity of Jersey milk collected throughout the lactation period.

Lactation Period and Season at Sampling	M1 September	M2 October	M4 December	M6 February	M8 April	M10 June	SE
Diversity indices							
Chao1	405 ^a^	313 ^ab^	270 ^ab^	388 ^a^	374 ^a^	217 ^b^	13.1
Shannon	6.01 ^a^	4.87 ^a^	3.56 ^b^	5.75 ^a^	5.17 ^a^	3.37 ^b^	0.12
Phyla/Families							
Proteobacteria	48.1 ^c^	59.1 ^bc^	74.3 ^ab^	47.2 ^c^	61.4 ^bc^	80.5 ^a^	1.44
Burkholderiaceae	22.4 ^cd^	12.2 ^d^	62.3 ^a^	12.2 ^d^	36.7 ^b^	29.2 ^bc^	0.97
Oxalobacteraceae	8.75 ^b^	0.87 ^b^	2.21 ^b^	1.71 ^b^	9.81 ^b^	41.4 ^a^	1.00
Bradyrhizobiaceae	3.31 ^a^	0.64 ^c^	2.09 ^ab^	0.53 ^c^	3.20 ^a^	0.96 ^bc^	0.10
Enterobacteriaceae	0.32 ^c^	36.8 ^a^	0.29 ^c^	25.8 ^b^	0.26 ^c^	4.88 ^c^	0.99
Moraxellaceae	1.95	3.84	1.14	2.80	1.67	0.76	0.26
Caulobacteraceae	3.84 ^ab^	0.16 ^d^	2.54 ^bc^	0.41 ^d^	4.05 ^a^	1.13 ^cd^	0.11
Firmicutes	33.6 ^a^	26.6 ^ab^	15.8 ^bc^	36.4 ^a^	23.4 ^abc^	12.6 ^c^	1.10
Erysipelotrichaceae	6.70 ^a^	4.66 ^ab^	2.72 ^ab^	5.14 ^ab^	4.26 ^ab^	1.90 ^b^	0.38
Ruminococcaceae	5.51	2.65	3.27	7.09	5.05	2.55	0.42
Lactobacillaceae	2.40	2.59	1.53	2.30	2.00	1.96	0.18
Streptococcaceae	1.26 ^b^	2.81 ^a^	0.75 ^b^	1.85 ^ab^	0.80 ^b^	0.51 ^b^	0.12
o_Clostridiales	2.35 ^a^	2.04 ^ab^	0.96 ^b^	2.54 ^a^	1.45 ^ab^	0.67 ^b^	0.12
Bacillaceae	0.81 ^b^	3.94 ^a^	0.06 ^b^	2.70 ^a^	0.13 ^b^	0.65 ^b^	0.13
Lachnospiraceae	2.45	1.04	1.61	4.25	3.26	0.80	0.34
Staphylococcaceae	3.27	2.32	1.49	3.10	1.40	0.52	0.34
Actinobacteria	5.16 ^a^	3.77 ^ab^	1.68 ^b^	3.25 ^ab^	2.64 ^ab^	1.38 ^b^	0.23
Bifidobacteriaceae	3.14	2.35	0.88	2.38	1.39	0.75	0.22
Bacteroidetes	9.19 ^a^	7.80 ^ab^	4.45 ^ab^	8.39 ^a^	5.91 ^ab^	3.09 ^b^	0.41
Bacteroidaceae	1.81 ^ab^	0.92 ^b^	0.96 ^b^	2.29 ^a^	1.26 ^ab^	0.66 ^b^	0.13
Others	0.59	0.83	1.50	2.46	3.56	1.30	0.17

Bacterial taxa with a relative abundance > 1% in at least one sample are indicated. Mean values for eight cows. Values in the same row with different superscript letters indicate statistically significant differences (*p* < 0.05). M1, M2, M4, M6, M8, and M10 represent the first, second, fourth, sixth, eighth, and tenth months of lactation, respectively.

## Data Availability

Raw data are stored in private computers and are available upon request.
